# The Prevalence of Microorganisms on Vegetables and Fruit from Wet Markets in Chiang Mai Province, Northern Thailand

**DOI:** 10.3390/foods15010080

**Published:** 2025-12-26

**Authors:** Sirikwan Dokuta, Sumed Yadoung, Phadungkiat Khamnoi, Sayamon Hongjaisee, Bajaree Chuttong, Surat Hongsibsong

**Affiliations:** 1 Clinical Laboratory, Research Institute for Health Sciences, Chiang Mai University, Chiang Mai 50200, Thailand; sirikwan.d@cmu.ac.th; 2 Environmental, Occupational Health Sciences and NCD Center of Excellence, Research Institute for Health Sciences, Chiang Mai University, Chiang Mai 50200, Thailand; summed.yadoung@cmu.ac.th; 3 Microbiology Unit, Diagnostic Laboratory, Maharaj Nakorn Chiang Mai Hospital, Faculty of Medicine, Chiang Mai University, Chiang Mai 50200, Thailand; phadungkiat.k@cmu.ac.th; 4 School of Health Sciences Research, Research Institute for Health Sciences, Chiang Mai University, Chiang Mai 50200, Thailand; sayamon.ho@cmu.ac.th; 5 Meliponini and Apini Research Laboratory, Department of Entomology and Plant Pathology, Faculty of Agriculture, Chiang Mai University, Chiang Mai 50200, Thailand; bajaree.c@cmu.ac.th

**Keywords:** antibiotic resistance, ESBL, Proteus, fruit, microorganism, opportunistic pathogen, vegetable, Chiang Mai, Thailand

## Abstract

Foodborne diseases remain a public health issue worldwide. Inadequate attention to food safety and hygiene increases the risk of opportunistic pathogens and resistant bacteria spreading to people through the food chain, leading to foodborne diseases. To investigate food safety in our region, this study aims to measure the prevalence of microorganisms on raw food materials randomly purchased from wet markets in Chiang Mai province, Northern Thailand. In this study, microbial cultures, identified by MALDITOF-MS techniques, were used to determine the microflora and antibiotic-resistance organisms on raw vegetables and fruit. Consequently, to confirm antibiotic resistance, the antimicrobial susceptibility techniques were performed. The results found no *Salmonella enterica* was detected on the overall food samples. For *Proteus* spp. detection, *P. mirabilis* were detected at 3.23% in cabbage, 3.57% in Chinese cabbage, and 6.67% in lettuce, while *P. vulgaris* were detected at 7.14% in Chinese cabbage and 3.57% in peppermint. No *Proteus* spp. was detected in basils, tomatoes and grapes. In addition, for antibiotic-resistance detection, only ESBL-producing *Klebsiella oxytoca* was detected in the raw tomato sample (3.57%). According to the study’s findings, people who participate in the food process should be aware of their food safety and hygiene.

## 1. Introduction

Foodborne disease is a major public health concern worldwide and can result in long-term impairment or death [1,2]. Currently, the prevalence of foodborne disease is increasing and putting a costly strain on healthcare systems. In 2015, the World Health Organization (WHO) released the first worldwide and regional evaluation of the illness burden related to 31 foodborne pathogens (bacteria, viruses, parasites, toxins, and chemicals) [3]. It emphasizes that foodborne illnesses can cause over 600 million cases and 420,000 deaths each year and result in a loss of 33 million healthy years of life, with the greatest cost being in low- and middle-income countries (LMIC). The most afflicted demographic group is children who are 5 years of age, which represents 40% of the foodborne disease burden, with 125,000 deaths per year [2]. Moreover, in the most recent data update in 2021, the global age-standardized disability-adjusted life years (DALYs) for enteric diseases were 1020.15 per 100,000 population [4].

According to the World Bank’s report in 2019, for the economic burden of foodborne diseases, the total production losses associated with these illnesses in low- and middle-income countries are estimated to be US $95.2 billion per year, with an annual cost of US $15 billion for disease treatment [5].

Foodborne diseases can arise at any point in the chain of food production, distribution, and consumption, as a result of food contamination. They can be caused by a variety of environmental contaminants, including air, water, and soil contamination, as well as inappropriate food preparation and storage [6].

Furthermore, climate change is projected to significantly impact food safety, potentially increasing the risk of existing and developing foodborne diseases through increased extreme weather events, rising air and water temperatures, and changes in rainfall frequency and intensity [7]. As a result, food contamination occurs when food becomes contaminated with foreign matter or substances by physical, chemical, or biological mechanisms [8].

Foodborne diseases cover a wide range of symptoms from diarrhea to cancer. The majority of symptoms are gastrointestinal infections; however, they can also have neurological, gynecological, and immunological symptoms. Nonetheless, there are three primary causes of illnesses: biological, chemical, and physical factors. Bacteria, viruses, and parasites are the most common biological threats that cause acute foodborne illnesses [9]. In addition to cancers, almost all of them are related to toxins or pathogens in contaminated food. One of the causes of cancer is mycotoxins, such as mold’s aflatoxins. These toxins are carcinogenic and have been connected to liver cancer. Therefore, prolonged exposure to other toxins can also raise the risk of cancer [10,11].

Currently, foodborne diseases are caused by different pathogens. *Salmonella* is one of the most common foodborne disease causes, accounting for 155,500 fatalities and 1.3 billion illnesses worldwide each year [12]. *Salmonella enterica*, a flagellated Gram-Negative bacillus, is a major cause of bacterial food poisoning worldwide. In the United States, an estimated 95% of human cases of Salmonellosis are caused by consuming contaminated food and beverages, and *Salmonella* is assumed to be responsible for 26% of all bacterial foodborne pathogen illnesses [10,13]. Meanwhile, in Asia, consumption of contaminated fresh produce, such as leafy greens, basil, sprouts, cucumber, and lettuce, has been related to a number of foodborne illness outbreaks caused by *S. enterica* [14,15].

Foodborne diseases are caused by *Proteus* species, which are widely spread in soil and water in a variety of ecological settings; eating contaminated meat, vegetables, or seafood might result in food poisoning [13,16]. Additionally, *Proteus* species are the most common cause of human urinary tract infections (UTIs). *Proteus* is responsible for 1% to 2% of all UTIs in otherwise healthy women and 5% of UTIs acquired in hospitals. At 20% to 45%, complicated UTIs (i.e., those caused by catheterization) are considerably more likely to be associated with *Proteus* infection [17,18].

For antimicrobial-resistant organism infection, exposure throughout the food chain has been linked to an increase in foodborne illness outbreaks in recent decades. Antimicrobial resistance (AMR) is one of the most serious global public health and development concerns. Bacterial AMR is expected to have contributed to 4.95 million fatalities worldwide in 2019 and to have been directly responsible for 1.27 million deaths [19]. Moreover, antibiotic abuse and misuse in veterinary and human medicine have been related to the emergence and spread of drug-resistant bacteria, making the treatment of infectious diseases in animals and humans ineffective [20].

Overall, *S. enterica*, *Proteus* species, and antibiotic-resistant pathogens were most significant contributors to foodborne illnesses globally. Nonetheless, Thailand has limited epidemiological data regarding these AMRs and contaminated pathogens. As a result, the purpose of this study is to use an appropriate analytical technique to detect the presence of pathogens in vegetable and fruit samples collected from wet markets in Chiang Mai province, Northern Thailand. In this study, samples of raw food materials with leafy vegetables and fruit were tested for contaminated organisms such as *S. enterica*, *Proteus* species, and antibacterial-resistant pathogens. To determine and confirm these dietary contaminants, microbial culture and identification were performed by the MALDI-TOF MS, VITEK^®^ MS machine [21,22,23]. Consequently, antimicrobial drug susceptibility testing was performed to confirm the suspected antibiotic-resistant organism. Thailand is a large producer of fresh fruits and vegetables, and a large portion of Thai food usually uses raw, fresh, or partially cooked vegetables, which may expose people directly to contaminated microorganisms, including foodborne AMR. We presume that the high prevalence of contaminated pathogens on fruit and vegetables may raise the risk of infection for people involved in the food chain. Early detection of foodborne microorganisms in food materials can help stop the spread of illnesses and avoid outbreaks and ensure that consumers obtain safe food, free of pathogens or food risks that can occur throughout the food chain. This challenge increases the responsibilities of food producers and operators to ensure consumer food safety.

## 2. Materials and Methods

### 2.1. Study Design

This cross-sectional study aimed to detect the contaminated microorganisms in leafy vegetable and fruit samples collected from 3 wet markets in Chiang Mai Province, Northern Thailand. These markets were chosen and studied because they have a large impact on many individuals in the city. All studied vegetables and fruits were popular raw food materials in our region. In this study, raw vegetable and fruit samples usually sold at markets in our area were randomly purchased and collected from each market between September 2023 and February 2024 (Table 1). All food material samples were tested for *S. enterica*, *Proteus* species, and antibiotic-resistant organisms included ESBL, CRE, MRSA, and VRE microbes by using microbial culture-based and identification techniques, and then antibiotic-resistant organisms were confirmed by antimicrobial susceptibility techniques.

### 2.2. Sample Collection

In total, the 7 food material items included raw leafy vegetables and fruit: basil, cabbage, Chinese cabbage, lettuce, peppermint, tomato, and grape were collected from 3 wet markets in Chiang Mai, Thailand. There were many shops in these markets. This study collected all the food items from each market by randomly collecting samples from the shops at each market. Each sample was stored in a separate labeled clean zip-lock bag. During transportation, all samples were kept in a cold container with the cold pack to control the low temperature between 4 and 8 °C. Consequently, all samples were transported to the microbiology laboratory as soon as possible or within 8 h. The total number of vegetable and fruit samples collected from each market is listed in Table 1.

### 2.3. Detection of Microorganism in Food Samples

#### 2.3.1. Microbial Culture Test (Culture-Based Method)

##### Detection of *S. enterica*

Sterile amies swabs were swabbed on the surface of 100 g of raw vegetable and fruit samples (used the new one for each sample), then swabbed in a round pattern on the modified semi-solid Rappaport Vassiliadis (MSRV) agar [24]. And then, all agar plates were incubated at 41.5 ± 1 °C, 18–24 h to allow bacterial cells to grow and replicate on the agar plate. Motile *Salmonella* can proliferate due to the medium’s low agar content, forming a distinctive opaque halo surrounding the inoculation site. For motile *Salmonella*, this swarming is a crucial sign of a successful test. After that, to subculture the motile *Salmonella*, a sterile inoculating loop was used to transfer a small amount of bacteria from the swarming area, then streak it out on the surface of the Salmonella Shigella agar (SS agar) for 3 plains for isolated colonies. Finally, this plate was incubated at 35 ± 2 °C, 18–24 h to provide bacterial cells replication.

##### Detection of Proteus Species

Sterile amies swabs were swabbed on the surface of 100 g of vegetable and fruit samples (we used a new one for each sample), then swabbed in a straight line (3–4 cm) on the surface of the blood agar (BA) plate, and then we incubated the plate at 35 ± 2 °C, 18–24 h to allow bacterial cells to grow and replicate on the agar plate. After incubation, the swarming motility that is characteristic of *Proteus* spp. was observed. For *Proteus* swarming identification, colonies showed up as thin, filmy layers in concentric circles and usually covered the entire plate. To subculture and isolate the *Proteus* organism, a sterile inoculating loop was used to transfer a small number of bacteria from thin and filmy colonies, then streaked out for isolated colonies on the surface of the MacConkey agar plate. Finally, this plate was incubated at 35 ± 2 °C, 18–24 h to provide bacterial cell replication.

##### Detection of Antibiotic-Resistant Microorganism

Sterile amies swabs were swabbed on the surface of 100 g of vegetable and fruit samples (used the new one for each sample), then swabbed in a round shape on the surface of selective CHROMID^®^ agar plates (bioMerieux SA, Marcy l’Etoile, France), including the CHROMID^®^ ESBL, CHROMID^TM^ CARBA, CHROMID^®^ MRSA SMART, and CHROMID^TM^ VRE agar plates, for the detection of ESBL-producing pathogens, CRE, MRSA, and VRE microorganisms, respectively [25,26]. And then, the sterile loop was used to streak and spread the sample on the surface of each agar plate in a zigzag pattern. After that, all selective agar plates were incubated at 35 ± 2 °C, 18–24 h to allow microbial cells to grow and replicate on the CHROMID^®^ agar plate.

For antibiotic resistant organism subculture and isolation, a sterile loop was used to bring a small amount of bacteria from the suspected colony with a specific color; the colony from the CHROMID^®^ ESBL and CHROMID^TM^ CARBA agar were subcultured and isolated on the MacConkey agar plate while the colonies from the CHROMID^®^ MRSA and CHROMID^TM^ VRE agar plates were subcultured and isolated on a Phenylethyl alcohol (PEA) agar plate. Finally, all plates were incubated at 35 ± 2 °C, 18–24 h to provide microbial cell replication.

##### Microbial Culture Test Quality Control (QC)

In this study, positive controls, which are well-characterized microorganisms with established growth patterns, are utilized to ensure that the system functions properly. Briefly, a sterile loop was used to transfer a small amount of positive control organism, which was subsequently streaked and disseminated over the surface of each media agar. For the swab sterility control test, new sterile amies swabs were swabbed onto the surface of each medium agar. For the culture media sterility control test, uninoculated medium plates were utilized to ensure that no contamination occurred during preparation, transportation, or storage. The QC process for all microbial culture tests was carried out as described above.

#### 2.3.2. Microbial Identification Test

To identify microorganism strains, the matrix-assisted laser desorption/ionization time-of-flight mass spectrometry (MALDI-TOF MS) machine was used in the process of the microbial identification test. The MALDI-TOF MS is an ionization technology that uses a laser energy-absorbing matrix to produce ions from large molecules with small fragmentation. This machine is a potent analytical method that quickly identifies microbes (bacteria, fungi) and characterizes biomolecules (proteins, peptides, lipids). In brief, the single colony of suspected organisms was mixed with a matrix solution on a target plate. Then, this mixture was further analyzed by the MALDI-TOF MS, VITEK^®^ MS (bioMerieux, Marcy l’Etoile, France) machine. In the machinery process, the mixture was struck with a laser to ionize and desorb the molecules and then they were measured by their flight time through a vacuum tube to determine their mass-to-charge ratio. Finally, the detected signal results were compared and evaluated with the machinery database to identify microorganism strains [22,23].

#### 2.3.3. Antimicrobial Susceptibility Test (AST)

In this study, the isolated colonies of suspected organisms were used to perform AST to determine and confirm the susceptibility or resistance of bacterial strains to different antibiotics. The standardized AST was performed by using the disk diffusion Kirby–Bauer technique with 0.5 McFarland turbidity standard methods, then the interpretation of the results was evaluated, followed by using the Clinical Laboratory Standards Institute (CLSI) guidelines, which is a standard protocol for AST [27].

Briefly, we suspended well-isolated suspected bacterial colonies into the broth culture to prepare the microbial suspension with 0.5 McFarland standard turbidity. Then, the sterile swab was used to bring and streak the suspension on the whole surface of the Mueller Hinton agar (MHA) plate. After that, the appropriated antibiotic disks of each microorganism were put on the surface of the MHA plate. Finally, all plates were incubated at 37 ± 2 °C, 18–24 h. For AST interpretation, the inhibition zone of each antibiotic disk on the cultured plate was determined to the nearest millimeter, and the result was evaluated to determine the susceptibility, intermediary, or resistance with a standard CLSI guideline.

In the process of AST by disk diffusion assay, antibiotic disks including Ceftazidime (CAZ30), Cefotaxime (CTX30), Ceftazidime/Clavulanic acid (CAC30/10), and Cefotaxime/Clavulanic acid (CEC30/10) were used to determine and confirm ESBL-producing antibiotic resistant bacteria. In a brief interpretation, the zones of inhibition were determined to the nearest millimeter, then the inhibition zone surrounding a cephalosporin disk (CAZ30 or CTX30) was compared to the zone of inhibition surrounding the same cephalosporin disk in combination with a beta-lactamase inhibitor, such as clavulanic acid (CAC30/10 or CEC30/10). An increase of ≥5 mm of the zone size with the addition of the inhibitor indicates the ESBL production [28].

As a reference, *E. coli* ATCC^®^ 25922, *E. coli* ATCC^®^ 35218 were used for quality control.

## 3. Results

### 3.1. Detection of S. enterica, Proteus Species, and Antibiotic-Resistant Microorganisms

The results of *S. enterica*, *Proteus* spp., and antibiotic-resistant organism detection in raw vegetables and fruit collected from three wet markets in Chiang Mai province between September 2023 and February 2024 are presented in Table 2 and Table 3. In this study, microbial culture and identification by the automated MALDI-TOF MS, VITEK^®^ MS (bioMerieux, Marcy l’Etoile, France) machine were used to detect these microorganisms in raw food material samples.

Among 203 samples, consisting of the following: 31 basil, 31 cabbage, 28 Chinese cabbage, 30 lettuce, 28 peppermint, 28 tomato, and 27 grape samples, no *S. enterica* was detected overall. For *Proteus* spp. detection, *P. mirabilis* was detected at 3.23% in cabbage, 3.57% in Chinese cabbage, and 6.67% in lettuce, while *P. vulgaris* was detected at 7.14% in Chinese cabbage and 3.57% in peppermint. In addition, no *Proteus* spp. were detected in basils, tomatoes, or grapes (Table 2).

For the antibiotic-resistant organism detection using the CHROMID^®^ selective agar plates (bioMerieux SA, Marcy l’Etoile, France), ESBL-producing *K. oxytoca* was only detected in the raw tomato sample (3.57%), whereas no ESBL, CRE, MRSA, or VRE-producing organisms were detected in the basil, cabbage, Chinese cabbage, lettuce, peppermint, or grape samples (Table 2). Nevertheless, other unexpected organisms were detected and isolated from these raw food samples and named in Table 3.

**Figure 1:**
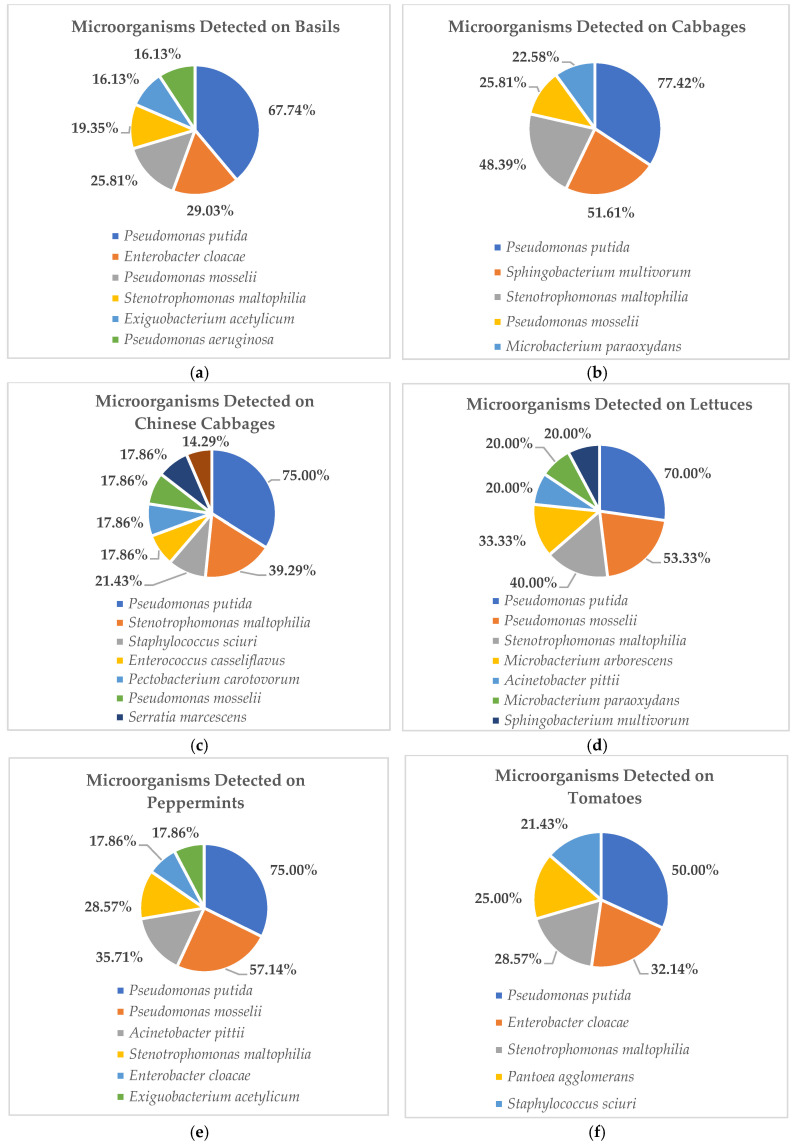

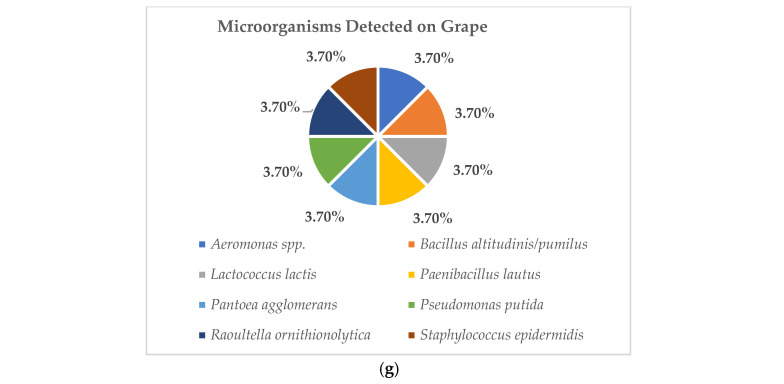
These figures show the 5 most prevalent microorganisms detected on raw vegetable and fruit samples obtained from wet markets in Chiang Mai Province between September 2023 and February 2024 (*n* = 203). (Figure 1a show the data of microorganism detected on basils. Figure 1b show the data of microorganism detected on cabbages. Figure 1c show the data of microorganism detected on Chinese cabbages. Figure 1d show the data of microorganism detected on lettuces. Figure 1e show the data of microorganism detected on peppermints. Figure 1f show the data of microorganism detected on tomatoes. Figure 1g show the data of microorganism detected on grapes.

Among the unexpected organisms detected, the five microorganisms most commonly found in raw vegetables are shown in Figure 1a–g. These data showed that *P. putida* was the most prevalent organism, detected on almost all collected vegetables, including basil, cabbage, Chinese cabbage, lettuce, peppermint, and tomato (Figure 1a–f), while on grapes, *P. putida* was detected at a similar rate to other organisms, which were eight organisms isolated from the 27 overall raw grape samples (Figure 1g). For further evaluation, the organisms detected from high to low prevalence on basils included *P. putida* (67.74%), *E. cloacae* (29.03%), *P. mosselii* (25.81%), *S. maltophilia* (19.35%), and *E. acetylicum* and *P. aeruginosa* (16.13%), respectively (Figure 1a). On cabbages, they were *P. putida* (77.42%), *S. multivorum* (51.61%), *S. maltophilia* (48.39%), *P. mosselii* (25.81%), and *M. paraoxydans* (22.58%), respectively (Figure 1b). For Chinese cabbages, they were *P. putida* (75.00%), *S. maltophilia* (39.29%), *S. sciuri* (21.43%), *E. casseliflavus*–*P. carotovorum–P. mosselii*, *S. marcescens* (17.86%), and *P. aeruginosa* (14.29%), respectively (Figure 1c). On lettuces, they were *P. putida* (70.00%), *P. mosselii* (53.33%), *S. maltophilia* (40.00%), *M. arborescens* (33.33%), and *A. pittii, M. paraoxydans, S. multivorum* (20.00%), respectively (Figure 1d). On peppermints, they were *P. putida* (75.00%), *P.* mosselii (57.14%), *A. pittii* (35.71%), *S. maltophilia* (28.57%), *E. cloacae*, and *E. acetylicum* (17.86%), respectively (Figure 1e). On tomatoes, they were *P. putida* (50.00%), *E. cloacae* (32.14%), *S. maltophilia* (28.57%), *P. agglomerans* (25.00%), and *S. sciuri* (21.43%), respectively (Figure 1f).

### 3.2. Antimicrobial Susceptibility Testing (AST)

In this study, AST was performed to determine and confirm antibiotic resistance from the isolated colonies of suspected organisms. In total, from 203 raw vegetable and fruit samples obtained from three wet markets in Chiang Mai province, the results found no antibiotic-resistant organism colonies related to the CRE, MRSA, and VRE detected on CHROMID^®^ CARBA, CHROMID^®^ MRSA SMART, and selective CHROMID^TM^ VRE agar plates (bioMerieux SA, Marcy l’Etoile, France), respectively. Nevertheless, only one isolated ESBL-producing *K. oxytoca* colony was detected from a raw tomato sample (1 of 28, 3.57%).

In addition, four colonies of *P. mirabilis* were isolated from cabbage, Chinese cabbage and lettuce, and three colonies of *P. vulgaris* were isolated from Chinese cabbage and peppermint. *Proteus* species are a Gram-Negative bacteria in the *Enterobacteriaceae* family. Therefore, the AST was performed to determine and confirm ESBL antibiotic drug susceptibility for all eight isolated colonies of Proteus and ESBL-producing *K. oxytoca*. In this study, AST was performed by Kirby–Bauer disk diffusion, specifically with the combined disk test with antibiotic disks CAZ30, CAC30/10, CTX30, and CEC30/10. Then, the results were interpreted according to the standard guidelines on antimicrobial susceptibility in the CLSI protocol. The study results found that only an isolated colony of ESBL-producing *K. oxytoka* was positive for ESBL, while other isolated colonies of *P. mirabilis* and *P. vulgaris* were drug-susceptible or negative for ESBL (Table 4).

## 4. Discussion

The prevalence of microorganisms on raw vegetables and fruit in Chiang Mai, Northern Thailand, has been a subject of concern due to food safety implications. Several studies have investigated bacterial contamination levels in these food items, highlighting the need for stringent food safety inspections. In this study, food safety was investigated. Raw edible leafy and skin vegetable and fruit samples were chosen to test the potential of microorganism contamination. Leafy vegetables play a significant role in the daily diet worldwide. Since they are low-calorie, high-nutrient sources of vitamins, minerals, fiber, and antioxidants that support bone and muscle health, they also prevent chronic diseases like cancer and cardiovascular conditions [29].

Nonetheless, if raw materials were contaminated with foreign matters, consuming leafy vegetables may considerably increase the potential pathogens that humans consume. The germs will multiply and release toxins that may impair the host’s physiological processes, leading to microbial foodborne diseases. Moreover, eating food that has previously been contaminated with bacterial endotoxins and exotoxins is another way to become exposed [30,31]. Typically, symptoms of foodborne diseases include fever, diarrhea, vomiting, nausea, and stomach discomfort, and more serious cases may result in systemic bacteremia and death [32,33]. Even though many countries have implemented integrated food safety monitoring systems, there is still a considerable risk of foodborne microorganisms spreading, especially for minimally processed foods such as raw food materials [34].

For the past, researchers have concentrated their efforts on studying the microbial contamination of minimally processed fruits and vegetables. Limited investigations have examined the isolation of bacteria from a variety of fruit- and vegetable-processing facilities, along with phenotypic antibiotic resistance evaluation. In this study, we investigated and provided the results of microorganism contamination and food safety in our community. We determined the prevalence of microorganism contamination on raw vegetables and fruit, such as basil, cabbage, Chinese cabbage, lettuce, peppermint, tomato, and grapes. All 203 raw food material samples were randomly purchased and collected from the shops in each wet market in Chiang Mai Province. These three markets were selected and used in the study because they have a significant impact on many people in the city. One of them is the biggest wholesale market, where food items and supplies are distributed around the city and the surrounding area, while the others are well-known and well-liked. As a result, the outcomes of this study may impact the local community.

The results of the contaminated microorganism detection in the microbial culture were identified by an automated MALDI-TOF MS, VITEK^®^ MS (bioMerieux, Marcy l’Etoile, France) machine technique. This study found no *S. enterica* or other CRE, MRSA, and VRE-producing antibiotic-resistant organisms detected on the overall raw vegetable and fruit samples. Besides other expected organism detection, some food samples contained *P. mirabilis*, *P. vulgaris*, and ESBL-producing *K. oxytoca*. The results demonstrated that no *Proteus* spp. was detected in basil, tomato, or grape, but *P. mirabilis* was the most detected in lettuce, followed by Chinese cabbage and cabbage, respectively, while *P. vulgaris* was only detected in Chinese cabbage and peppermint (Table 2). Several studies reported that *Proteus* spp. can cause food poisoning and most of them are usually found and isolated from retail meats, aquatic products, and carcasses [35,36,37].

The antibiotic-resistant microorganism detection results from the CHROMID^®^ selective agar (bioMerieux SA, Marcy l’Etoile, France) were confirmed with a microbial antibiotic drug susceptibility test. Only ESBL-producing *K. oxytoca* was found in the raw tomato sample. As previously reported, this isolated ESBL-producing organism was confirmed and detected with the *bla*_CTX-M_ gene, which represented the ESBL-producing gene [26], whereas no ESBL, CRE, MRSA, or VRE-producing organisms were detected in other vegetable and fruit samples (Table 2). According to several studies, *K. oxytoca* can contaminate raw vegetables, fruits, and water, and it often contaminates via fecal matter, improper handling, or cross-contamination during storage. *Klebsiella oxytoca* is an opportunistic pathogen that is normally found in the gut but can cause infections in immunocompromised patients, such as urinary tract infections (UTIs), bacteremia, and wound and respiratory infections. ESBL-producing *K. oxytoca* is a clinically significant bacterium because it carries ESBLs—enzymes that can break down a wide range of beta-lactam antibiotics, including penicillin and third-generation cephalosporin, making infections harder to treat. ESBL-producing *K. oxytoca* is resistant to many antibiotics, limiting treatment options for carbapenems or other advanced antimicrobials [38].

Nevertheless, although no other antibiotic-drug-resistant pathogens were detected in this study, numerous and diverse opportunistic organisms were determined from studying the raw vegetable and fruit samples (Table 3) and the five most frequently detected microbes are shown in Figure 1a–g. The results showed that nearly all of them were opportunistic pathogens belonging to the following genera: *Pseudomonas, Enterobacter, Enterococcus, Exiguobacterium, Microbacterium, Staphylococcus, Sphingobacterium,* and *Stenotrophomonas*, whereas *P. putida* was the most dominant detected among these high-prevalence organisms. In this study, *P. putida* were detected from high to low prevalence at 77.42% on cabbages, 75.00% on Chinese cabbages and peppermints, 70.00% on lettuces, 67.74% on basils, and 50.00% on tomatoes, respectively. From our findings, all obtained pathogens can impact human health problems due to their potential risk for opportunistic infections such as pneumonia, fungal thrush, and some bacterial infections. Opportunistic infections affect people with compromised immune systems more frequently or more severely than people with strong immune systems. Symptoms of this infection include persistent fever, chills, night sweats, unexplained weight loss, swollen lymph nodes, loss of appetite, etc. [39,40]. Nevertheless, several studies have reported almost all of the opportunistic pathogens that are primarily found in temperate soil and water environments [41,42,43,44].

According to multiple studies linked to foodborne pathogen-caused disease outbreaks, such as salmonellosis, listeriosis, and campylobacteriosis, one of the main things that causes pathogens to enter water is the source of irrigation water, such as surface water, groundwater, treated sewage, or reservoir water [45,46,47]. In addition, direct zoonotic contamination from intense and vast livestock production facilities near the crop site may contribute to pre-harvest contamination. Microorganisms can also be transferred to raw material due to the occurrence of wild animals and crop pests, such as insects, rodents, and birds [48,49,50]. Furthermore, fruits and vegetables can be contaminated by microorganisms during the fresh-cut processing and post-harvest stage, such as when field workers do not follow proper hygiene procedures or when tools and machinery used to harvest raw materials are not properly maintained, preserved, or transported. Moreover, variable factors pertaining to geographic location, the type of raw material treated, technological process, disinfection method, season, etc., account for variations in microbiological contamination levels [51,52,53,54,55].

According to our findings, foodborne pathogens in raw leafy vegetables and fruit may impact people who work in the food industry in our region. The handling and consumption of raw cabbage, Chinese cabbage, lettuce, and peppermint may infect them with Proteus infections. Raw tomatoes have the potential to cause an antibiotic-resistant *K. oxytoca* infection. All of the foods collected in this investigation have the potential to cause opportunistic infections. The study results from Beijing, China reported that 86 microorganism stains were detected from 326 fresh-cut fruit and vegetable samples, with the highest prevalence being *Staphylococcus aureus* at 15.38%, followed by *E. coli* at 9.23% and *Listeria monocytogenes* at 1.85%, while no *Salmonella* was detected [56]. According to data from the United States, fruits and vegetables account for approximately 46% of foodborne infections, with norovirus, *Salmonella* spp., and *E. coli* O157:H7 being the most common carriers [57].

For the limitations of this study, due to the study aiming to determine the prevalence and the presence of contaminated microorganisms in raw vegetable and fruit samples obtained from locally wet markets, we only performed a microbiological qualitative method with microbial culture identification by the MALDI-TOF MS, VITEK^®^ MS machine to investigate them. In microbiology, qualitative methods are used to observe the characteristics of isolated microorganisms, allowing us to identify them or, more frequently, to detect the presence of organisms of interest or concern. Therefore, we did not quantify the microbial contamination as CFU/g (colony forming units per gram), which is the microbiological quantitative method [58]. Moreover, in this study, the mass spectrometry technique was used to identify bacterial species of the isolated strains. The matrix-assisted laser desorption–ionization time of flight mass spectrometry (MALDI-TOF MS) machines are mostly used in clinical microbiological investigations [22], which reveal the existence of bacteria from a variety of sources, including plant, animal, and human pathogenic bacteria, soil bacteria, and signs of fecal contamination [25,26,59]. Nonetheless, because of the limitation of this machinery evaluation technique, some isolated organisms were unable to be identified in this study. For further studies, high-sensitivity and specific approaches such as molecular identification methods will be applied to overcome this challenge.

Overall, our findings on the prevalence of microbial contamination of raw vegetables and fruit in our area may serve to recognize and direct future efforts to improve food production safety and hygiene. Some isolated organisms are potentially dangerous, endangering public health. Food safety is the process of regulating food across the food chain, from production to consumption, to guarantee that it is free of biological pathogens, chemicals, and physical hazards. Personal hygiene, temperature control, surface cleaning, and chemical management are key principles. This protects consumers from foodborne illnesses, increases confidence, and encourages sustainable food enterprises. It is necessary to emphasize to local people the need to adhere to strict sanitary standards and improve our understanding of microbial contamination in raw food materials.

## 5. Conclusions

In this study, opportunistic pathogens and ESBL-producing antibiotic resistant organisms were determined to be on raw vegetables and fruit that were randomly obtained from wet markets in Chiang Mai province, Northern Thailand. The results highlight the increased risk of infection with antibiotic resistance and foodborne microorganisms among those involved in the food chain, such as harvesting, processing, shipping, and storage. These findings emphasize the need to monitor microbial microflora in raw food materials, as well as the requirement for adequate sanitary control techniques to reduce the risk of microorganism contamination and ensure that materials stay safe and of high quality throughout the supply chain.

## Figures and Tables

**Table 1:** Total number of vegetable and fruit samples collected from 3 wet markets in Chiang Mai province, Thailand, during September 2023–February 2024, *n* = 203.

Type of Samples	Number of Food Material Samples Collected			
	Market 1	Market 2	Market 3	Total (*n* = 203)
Basil	9	13	9	31
Cabbage	9	13	9	31
Chinese Cabbage	9	10	9	28
Lettuce	9	12	9	30
Peppermint	9	10	9	28
Tomato	9	10	9	28
Grape	9	9	9	27

**Table 2:** The results of microorganisms detected on raw vegetable and fruit samples.

Type of Food Material Samples (*n* = 203)	Results of Microorganism Detected on Raw Vegetable and Fruit Samples Identified by MALDI-TOF MS (VITEK MS, bioMerieux, Marcy-l’Étoile, France)					
	ESBL	CRE	MRSA	VRE	*S. enterica*	*Proteus* spp.
Basil (*n* = 31)	Not detected (0%)	Not detected (0%)	Not detected (0%)	Not detected (0%)	Not detected (0%)	Not detected (0%)
Cabbage (*n* = 31)	Not detected (0%)	Not detected (0%)	Not detected (0%)	Not detected (0%)	Not detected (0%)	***P. mirabilis*** ** = 3.23%**
Chinese Cabbage (*n* = 28)	Not detected (0%)	Not detected (0%)	Not detected (0%)	Not detected (0%)	Not detected (0%)	***P. mirabilis*** ** = 3.57%** ***P. vulgaris*** ** = 7.14%**
Lettuce (*n* = 30)	Not detected (0%)	Not detected (0%)	Not detected (0%)	Not detected (0%)	Not detected (0%)	***P. mirabilis*** ** = 6.67%**
Peppermint (*n* = 28)	Not detected (0%)	Not detected (0%)	Not detected (0%)	Not detected (0%)	Not detected (0%)	***P. vulgaris*** ** = 3.57%**
Tomato (*n* = 28)	***K. oxytoca*** ** = 3.57%**	Not detected (0%)	Not detected (0%)	Not detected (0%)	Not detected (0%)	Not detected (0%)
Grape (*n* = 27)	Not detected (0%)	Not detected (0%)	Not detected (0%)	Not detected (0%)	Not detected (0%)	Not detected (0%)

**Table 3:** The prevalence of microorganisms detected on vegetable and fruit samples.

Type of Food Material Samples	Results of Microorganisms Detected on Raw Vegetable and Fruit Samples Identified by MALDI-TOF MS (VITEK^®^ MS, bioMerieux, Marcy-l’Étoile, France)		
Basil (*n* = 31)	*Acinetobacter baumannii *(9.68%) *Acinetobacter pittii *(12.90%) *Acinetobacter radioresistens *(3.23%) *Aerococcus viridans *(3.23%) *Aeromonas punctata (caviae)* (9.68%) *Bacillus altitudinis/pumilus *(6.45%) *Bacillus cereus* gr. (6.45%) *Bacillus megaterium *(3.23%) *Chryseobacterium* spp. (3.23%) *Chryseobacterium gleum *(3.23%) *Chryseobacterium indologenes *(3.23%) *Comamonas testosterone *(3.23%) *Corynebacterium glutamicum *(3.23%) *Elizabethkingia anopheles *(3.23%) *Empedobacter brevis *(12.90%) *Enterobacter* spp. (9.68%) *Enterobacter cancerogenus *(3.23%) *Enterobacter cloacae *(29.03%)	*Enterobacter hormaechei* (9.68%) *Enterobacter kobei *(3.23%) *Enterobacter ludwigii* (3.23%) *Enterococcus casseliflavus *(6.45%) *Enterococcus faecalis* (9.68%) *Enterococcus gallinarum* (3.23%) *Exiguobacterium acetylicum *(16.13%) *Franconibacter pulveris *(3.23%) *Herbaspirillum huttiense *(3.23%) *Klebsiella aerogenes *(3.23%) *Klebsiella oxytoca *(3.23%) *Klebsiella pneumoniae *(12.90%) *Lactococcus lactis *(9.68%) *Leclercia adecarboxylata *(3.23%) *Leuconostoc lactis *(3.23%) *Listeria welshimeri *(3.23%) *Microbacterium paraoxydans *(9.68%) *Pantoea agglomerans *(12.90%)	*Pantoea dispersa *(9.68%) *Pectobacterium carotovorum *(9.68%) *Pseudomonas* spp. (12.90%) *Pseudomonas aeruginosa *(16.13%) *Pseudomonas mendocina *(3.23%) *Pseudomonas mosselii *(25.81%) *Pseudomonas putida *(67.74%) *Raoultella ornithionolytica *(3.23%) *Serratia marcescens *(3.23%) *Sphingobacterium multivorum *(6.45%) *Sphingobacterium thalpophilum *(3.23%) *Staphylococcus gallinarum *(6.45%) *Staphylococcus sciuri *(3.23%) *Staphylococcus xylosus *(3.23%) *Stenotrophomonas maltophilia *(19.35%) *Vagococcus fluvialis *(3.23%) *Weissella confusa *(12.90%)
Cabbage (*n* = 31)	*Achromobacter xylosoxidans *(3.23%) *Acinetobacter baumannii *(3.23%) *Acinetobacter gyllenbergii *(3.23%) *Acinetobacter pittii *(19.35%) *Aeromonas* spp. (3.23%) *Aeromonas punctata (caviae) *(3.23%) *Bacillus thermoamylovorans *(3.23%) *Cedecea lapagei *(6.45%) *Cedecea neteri *(3.23%) *Chryseobacterium* spp. (3.23%) *Chryseobacterium gleum *(16.13%) *Chryseobacterium indologenes *(19.35%) *Elizabethkingia anopheles *(3.23%) *Empedobacter brevis *(3.23%) *Enterococcus casseliflavus *(3.23%) *Enterobacter cloacae *(6.45%)	*Exiguobacterium acetylicum *(6.45%) *Klebsiella oxytoca *(3.23%) *Klebsiella pneumoniae *(6.45%) *Klebsiella variicola *(3.23%) *Lactococcus garvieae *(3.23%) *Lactococcus lactis *(3.23%) *Leclerica adecarboxylata *(3.23%) *Microbacterium* spp. (3.23%) *Microbacterium arborescens *(19.35%) *Microbacterium oxydans *(9.68%) *Microbacterium paraoxydans *(22.58%) *Microbacterium testaceum *(9.68%) *Myroides* spp. (9.68%) *Myroides marinus *(6.45%) *Pantoea agglomerans *(3.23%) *Pectobacterium carotovorum *(9.68%)	*Pseudomonas* spp. (9.45%) *Pseudomonas aeruginosa *(3.23%) *Pseudomonas chlororaphis *(3.23%) *Pseudomonas putida *(77.42%) *Pseudomonas mendocina *(3.23%) *Pseudomonas mosselii *(25.81%) *Rhizobium radiobacter *(6.45%) *Rothia kristinae *(9.68%) *Serratia marcescens *(6.45%) *Sphingobacterium multivorum *(51.61%) *Staphylococcus hominis *(3.23%) *Staphylococcus saprophyticus *(3.23%) *Staphylococcus sciuri *(12.90%) *Staphylococcus xylosus *(3.23%) *Stenotrophomonas maltophilia *(48.39%) *Weissella confusa *(6.45%)
Chinese Cabbage (*n* = 28)	*Acinetobacter baumannii *(3.57%) *Acinetobacter calcoaceticus *(7.14%) *Acinetobacter lwoffii *(10.71%) *Acinetobacter pittii *(10.71%) *Aeromonas hydrophilia *(3.57%) *Aeromonas media *(3.57%) *Bacillus cereus* gr. (3.57%) *Bacillus megaterium *(3.57%) *Chryseobacterium indologenes *(3.57%) *Comamonas testosterone *(3.57%) *Empedobacter brevis *(7.14%) *Enterobacter asburiae *(3.57%) *Enterobacter cloacae *(10.71%) *Enterococcus casseliflavus *(17.86%) *Enterococcus mundtii *(3.57%)	*Exiguobacterium acetylicum *(10.71%) *Exiguobacterium aurantiacum *(3.57%) *Finegoldia magna *(3.57%) *Herbaspirillum huttiense *(3.57%) *Klebsiella aerogenes *(3.57%) *Klebsiella oxytoca *(7.14%) *Klebsiella pneumoniae *(7.14%) *Lactococcus garvieae *(3.57%) *Lactococcus lactis *(3.57%) *Leclercia adecarboxylata *(3.57%) *Microbacterium arborescens *(3.57%) *Microbacterium paraoxydans *(7.14%) *Myroides* spp. (7.14%) *Pectobacterium carotovorum *(17.86%) *Pseudomonas* spp. (10.71%)	*Pseudomonas aeruginosa *(14.29%) *Pseudomonas putida *(75.00%) *Pseudomonas mosselii *(17.86%) *Pseudomonas nitroreducens *(3.57%) *Pseudomonas straminea *(3.57%) *Raoultella ornithionolytica *(7.14%) *Serratia marcescens *(17.86%) *Sphingobacterium multivorum *(10.71%) *Staphylococcus aureus *(3.57%) *Staphylococcus sciuri *(21.43%) *Staphylococcus xylosus *(10.71%) *Stenotrophomonas maltophilia *(3.57%) *Weissella confusa *(21.43%)
Lettuce (*n* = 30)	*Acinetobacter baumannii *(10.00%) *Acinetobacter calcoaceticus *(6.67%) *Acinetobacter nosocomialis *(3.33%) *Acinetobacter pittii *(20.00%) *Aeromonas punctata (caviae) *(10.00%) *Aeromonas sobria *(3.33%) *Bacillus cereus* gr. (6.67%) *Bacillus megaterium *(3.33%) *Bergeyella zoohelcum *(3.33%) *Cedecea lapagei *(3.33%) *Citrobacter koseri *(3.33%) *Chryseobacterium* spp. (16.67%) *Chryseobacterium gleum *(3.33%) *Chryseobacterium indologenes *(10.00%) *Elizabethkingia anopheles *(16.67%) *Empedobacter brevis *(6.67%) *Enterobacter cloacae *(16.67%)	*Escherichia coli *(3.33%) *Exiguobacterium acetylicum *(13.33%) *Klebsiella pneumoniae *(13.33%) *Kluyvera ascorbate *(3.33%) *Lactococcus lactis *(3.33%) *Microbacterium arborescens *(33.33%) *Microbacterium paraoxydans *(20.00%) *Myroides* spp. (10.00%) *Myroides marinus *(3.33%) *Paenibacillus lautus *(3.33%) *Pantoea agglomerans *(10.00%) *Pantoea ananatis *(3.33%) *Pectobacterium carotovorum *(3.33%) *Pseudomonas* spp. (10.00%) *Pseudomonas aeruginosa *(6.67%) *Pseudomonas alcaligenes *(3.33%) *Pseudomonas fluorescens *(6.67%)	*Pseudomonas otitidis *(3.33%) *Pseudomonas putida *(70.00%) *Pseudomonas mosselii *(53.33%) *Pseudomonas oleovorans *(3.33%) *Pseudomonas oryzihabitans *(3.33%) *Pseudomonas viridiflava *(3.33%) *Raoultella ornithionolytica *(3.33%) *Rhizobium radiobacter *(3.33%) *Sphingobacterium multivorum *(20.00%) *Sphingomonas parapaucimobilis *(6.67%) *Sphingobacterium thalpophilum *(6.67%) *Staphylococcus arlettae *(3.33%) *Staphylococcus sciuri *(3.33%) *Stenotrophomonas maltophilia *(40.00%) *Weissella confusa *(6.67%)
Peppermint (*n* = 28)	*Acinetobacter baumannii *(14.29%) *Acinetobacter gyllenbergii *(3.57%) *Acinetobacter pittii *(35.71%) *Aerococcus viridans *(3.57%) *Aeromonas hydrophilia *(3.57%) *Aeromonas punctata (caviae) *(7.14%) *Bacillus cereus* gr. (14.29%) *Bacillus megaterium *(7.14%) *Cedecea lapagei *(3.57%) *Chryseobacterium gleum *(7.14%) *Comamonas testosterone *(3.57%) *Corynebacterium glutamicum *(3.57%) *Delftia acidovoran *(3.57%) *Elizabethkingia anopheles *(3.57%) *Empedobacter brevis *(7.14%)	*Enterobacter asburiae *(7.14%) *Enterobacter cloacae *(17.86%) *Enterococcus casseliflavus *(3.57%) *Exiguobacterium acetylicum *(17.86%) *Escherichia coli *(3.57%) *Klebsiella aerogenes *(3.57%) *Klebsiella oxytoca *(3.57%) *Klebsiella pneumoniae *(3.57%) *Kocuria palustris *(3.57%) *Lactococcus lactis *(3.57%) *Microbacterium paraoxydans *(7.14%) *Myroides* spp. (3.57%) *Myroides ordoratus *(3.57%) *Pectobacterium carotovorum *(14.29%) *Pseudomonas* spp. (7.14%)	*Pseudomonas alcaligenes *(3.57%) *Pseudomonas aeruginosa *(10.71%) *Pseudomonas putida *(75.00%) *Pseudomonas mendocina *(3.57%) *Pseudomonas mosselii *(57.14%) *Raoultella ornithionolytica *(7.14%) *Raoultella terrigena *(3.57%) *Serratia marcescens *(3.57%) *Staphylococcus kloosii *(3.57%) *Staphylococcus sciuri *(7.14%) *Staphylococcus xylosus *(3.57%) *Stenotrophomonas maltophilia *(28.57%) *Weissella confusa *(14.29%)
Tomato (*n* = 28)	*Acinetobacter baumannii *(10.71%) *Acinetobacter pittii *(10.71%) *Bacillus cereus* gr. (10.71%) *Bacillus altitudinis/pumilus *(10.71%) *Bacillus subtilis/amyloliquefaciens/vallismortis *(3.57%) *Cedecea* spp. (3.57%) *Citrobacter freundii *(3.57%) *Chryseobacterium indologenes *(3.57%) *Empedobacter brevis *(3.57%) *Enterobacter asburiae *(3.57%) *Enterobacter cloacae *(32.14%) *Enterobacter hormaechei *(3.57%) *Enterococcus* spp. (3.57%) *Enterococcus faecalis *(3.57%) *Exiguobacterium acetylicum *(3.57%)	*Helicobacter pylori *(3.57%) *Klebsiella oxytoca *(14.29%) *Klebsiella pneumoniae *(7.14%) *Lactococcus lactis *(17.86%) *Microbacterium arborescens *(3.57%) *Microbacterium paraoxydans *(3.57%) *Paenibacillus lautus *(3.57%) *Paenibacillus pabuli *(3.57%) *Pantoea agglomerans *(25.00%) *Pantoea ananatis *(3.57%) *Pluralibacter gergoviae *(3.57%) *Providencia rettgeri *(3.57%) *Pseudomonas aeruginosa *(10.71%) *Pseudomonas otitidis *(3.57%) *Pseudomonas putida *(50.00%)	*Pseudomonas oryzihabitans *(3.57%) *Pseudomonas straminea *(3.57%) *Serratia rubidaea *(10.71%) *Sphingobacterium multivorum *(14.29%) *Sphingobacterium thalpophilum *(3.57%) *Staphylococcus ariettae *(3.57%) *Staphylococcus cohnii *(3.57%) *Staphylococcus gallinarum *(3.57%) *Staphylococcus haemolyticus *(10.71%) *Staphylococcus saprophyticus *(3.57%) *Staphylococcus sciuri *(21.43%) *Staphylococcus xylosus *(14.29%) *Stenotrophomonas maltophilia *(28.57%) *Weissella confusa *(14.29%)
Grape (*n* = 27)	*Aeromonas* spp. (3.70%) *Bacillus altitudinis/pumilus *(3.70%) *Lactococcus lactis *(3.70%)	*Paenibacillus lautus *(3.70%) *Pantoea agglomerans* (3.70%) *Pseudomonas putida *(3.70%)	*Raoultella ornithionolytica *(3.70%) *Staphylococcus epidermidis *(3.70%)

**Table 4:** Antimicrobial susceptibility testing (AST) results of microorganisms isolated from raw vegetables collected from 3 wet markets in Chiang Mai province, Northern Thailand between September 2023 and February 2024.

Colony No.	Microorganism	Source of Microorganism	Antibiotic Inhibition Zone Size (mm.)				ESBL-AST Interpretation *
			CAZ30	CAC30/10	CTX30	CEC30/10	
1	*K. oxytoca*	Tomato	25	22	**21**	**29**	**Positive**
2	*P. mirabilis*	Cabbage	26	26	31	31	Negative
3	*P. mirabilis*	Chinese cabbage	25	24	35	35	Negative
4	*P. mirabilis*	Lettuce	22	21	30	32	Negative
5	*P. mirabilis*	Lettuce	28	27	34	33	Negative
6	*P. vulgaris*	Chinese cabbage	21	20	25	24	Negative
7	*P. vulgaris*	Chinese cabbage	27	25	30	31	Negative
8	*P. vulgaris*	Peppermint	22	19	23	25	Negative

Remark: * The interpretation of the ESBL positive result or ESBL resistance is an increase in the zone of inhibition around the disk with clavulanic acid compared to the disk without clavulanic acid (typically ≥5 mm increase) [28].

## Data Availability

The datasets used and/or analyzed during the current study are available from the corresponding author upon reasonable request.
